# Successful Surgical Treatment in Empyema Caused by an Atypical Pathogen: Non-typhoid Salmonella

**DOI:** 10.7759/cureus.101725

**Published:** 2026-01-17

**Authors:** Yhonatan R Ramirez-Guerra, Sheccid J Enriquez, Adolfo A Echavarría, Jorge I Leyva Villegas, Gerardo E Muñoz-Maldonado

**Affiliations:** 1 General Surgery, Hospital Universitario "Dr. José Eleuterio González", Monterrey, MEX; 2 Cardiothoracic Surgery, Hospital Universitario "Dr. José Eleuterio González", Monterrey, MEX

**Keywords:** non-typhoidal salmonella (nts), salmonella infection, thoracic empyema, thoracotomy procedure, video-assisted thoracoscopic surgery (vats)

## Abstract

Empyema is most often associated with bacterial pneumonia, with *Streptococcus pneumoniae* being the predominant pathogen. *Salmonella* species are a rare cause of pleural empyema, typically occurring in immunocompromised individuals. We report a case of *Salmonella enteritidis* empyema in a 16-year-old male with a history of bronchial asthma but no other known risk factors. The patient presented with a 10-day history of right-sided pleuritic chest pain, fever, and dyspnea. Imaging revealed a complex right-sided pleural effusion with an air-fluid level. Initial thoracentesis was unsuccessful, and a chest tube drained hematopurulent fluid. Despite broad-spectrum antibiotics, clinical response was inadequate, prompting surgical intervention. Due to intraoperative bronchospasm, video-assisted thoracoscopic surgery was converted to open thoracotomy with pleurectomy. Cultures from the pleural fluid identified *S. enteritidis*. The patient recovered well and was discharged on oral antibiotics. Follow-up confirmed full clinical and radiological resolution. This case highlights the need to consider *Salmonella* as a potential cause of empyema even in immunocompetent adolescents. Early surgical intervention may be essential in refractory cases. Asthma, although not classically immunosuppressive, may predispose patients to invasive infections and warrants further investigation as a potential risk factor.

## Introduction

Empyema is defined as the accumulation of purulent fluid within the pleural space. Approximately 74% of cases are associated with parapneumonic effusions. The condition is typically bacterial in origin, with *Streptococcus pneumoniae* being the most implicated organism. Clinical manifestations of empyema typically include fever, dyspnea, cough, and pleuritic chest pain. Management relies on the prompt initiation of antibiotics and efficient pleural drainage. While tube thoracostomy serves as an initial intervention, more invasive procedures, such as video-assisted thoracoscopic surgery (VATS) or open thoracotomy, are often required to ensure the complete resolution of purulent collections and allow for adequate lung re-expansion [1].

*Salmonella *infections range from gastroenteritis to systemic sepsis, depending on the serotype and the patient's immune status. Typhoidal strains (*Salmonella *typhi and *Salmonella* paratyphi) are associated with systemic enteric fever, whereas non-typhoidal strains generally manifest as localized gastroenteritis, though they possess the potential for extraintestinal dissemination in susceptible populations [2].

Empyema secondary to *Salmonella *species is rare. Risk factors predisposing to *Salmonella*-related pleuropulmonary infections include immunocompromising conditions, such as diabetes mellitus, chronic alcohol use, sickle cell disease, malignancies, HIV infection, and malnutrition [3].

We report a rare case of empyema caused by *Salmonella *enteritidis in a previously healthy adolescent with bronchial asthma, successfully treated with surgical intervention.

## Case presentation

A 16-year-old male with a history of bronchial asthma, managed with budesonide and salbutamol, presented to the emergency department with a 10-day history of right-sided pleuritic chest pain. He had been hospitalized for an asthma exacerbation three months prior. An initial assessment by an external physician resulted in empiric treatment with ceftriaxone and levofloxacin, due to high suspicion of community-acquired pneumonia. Following a chest radiograph that revealed a right-sided pleural effusion, the patient was subsequently referred to our institution.

On admission, the patient reported fever and dyspnea for five days. Vital signs revealed a temperature of 38.0°C, tachycardia, and normal oxygen saturation. Physical examination showed asymmetry of the thorax, decreased breath sounds on the right side, and crepitant rales. Relevant laboratory findings are shown in Table 1.

**Table 1:** Laboratory findings at admission. PaO₂: partial pressure of oxygen in venous blood; PaCO₂: partial pressure of carbon dioxide in venous blood; HCO₃⁻: bicarbonate

Parameter	Value	Reference range
Hemoglobin (g/dL)	13.10	12.20-18.10
White blood cells (10^3^/μL)	18.10	4.0-11.0
Platelets (10^3^/μL)	481	142.0-424.0
Prothrombin time (seconds)	16.20	10.43-12.80
Activated partial thromboplastin time (seconds)	34.9	25.1-36.0
International normalized ratio (INR)	1.35	-
pH	7.42	7.35-7.45
PaO_2_ (mmHg)	15	80-100
PaCO_2_ (mmHg)	38	35-45
HCO_3_ (mmol/L)	24.3	21-28
Lactate (mmol/L)	2.1	0.5-2.2
CRP (mg/dL)	24.60	0.00-1.00

**Figure 1:**
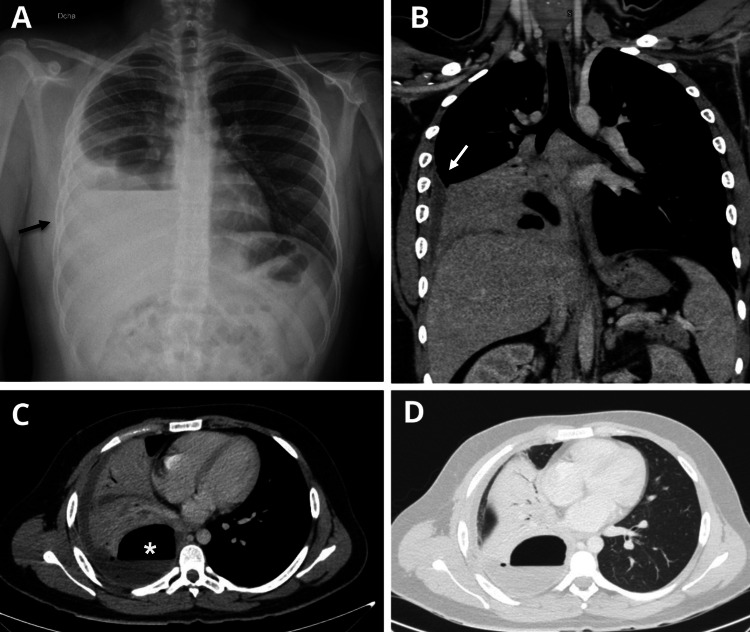
Chest radiograph showing unilateral right pleural effusion with a lenticular shape (A). In image (B), CT in the coronal view observing complex pleural effusion with pleural enhancement, laminar atelectasis (C), and empyema predominantly in the right middle lobe, occupying approximately 30% of the hemithorax (C and D), along with evidence of lower lobe pneumonia. CT: Computed tomography The black arrow shows pleural effusion on the right hemithorax in the chest radiograph. The white arrow indicates pleural enhancement. The white asterisk shows the air-fluid level on the right side of the chest.

Upon presentation, the chest X-ray demonstrated a persistent right-sided pleural effusion with an air-fluid level (Figure 1A). CT imaging of the chest confirmed a complex pleural effusion with pleural enhancement, laminar atelectasis, and empyema predominantly in the right middle lobe, occupying approximately 30% of the hemithorax, along with evidence of lower lobe pneumonia (Figures 1B-1D).

Initial thoracentesis was unsuccessful in evacuating the effusion, prompting the placement of a right-sided chest tube, which drained 20 cc of hematopurulent material.

Empiric antibiotic therapy was initiated with piperacillin-tazobactam 4 g IV every six hours. Due to persistent fever, leukocytosis, and inadequate clinical response, therapy was escalated to meropenem 1 g IV every eight hours and vancomycin 1.7 g IV every eight hours.

After five days of broad-spectrum antibiotics and chest tube drainage with no improvement, surgical intervention was pursued. During selective intubation for VATS, the patient experienced severe bronchospasm and oxygen desaturation, requiring bronchodilators with adequate response, and decided to convert to open right posterolateral thoracotomy. Intraoperatively, multiple loculated collections were identified in the right middle and lower lobes, along with pleural thickening (up to 1 cm). A pleurectomy was performed with release of the inferior pulmonary ligament, evacuating approximately 250 cc of hematopurulent material, with satisfactory re-expansion of the right lung confirmed intraoperatively and on postoperative imaging.

**Figure 2:**
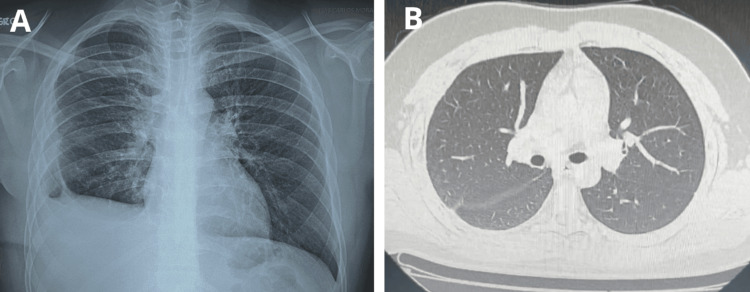
Chest X-ray showing adequate lung re-expansion and elevation of right hemidiaphragm (A). CT image in the lung window with resolution of empyema with good response and lung re-expansion (B).

Following surgery, the patient experienced significant clinical improvement. Meropenem monotherapy was continued for a total of seven days. *Salmonella *enteritidis was isolated from the pleural fluid culture taken during the surgical procedure, prompting a switch to oral trimethoprim-sulfamethoxazole (160/800 mg) twice daily, which was continued after discharge. Due to the unusual bacterial etiology, a fourth-generation ELISA for HIV was performed, and it was negative. At the 30-day follow-up, the patient was asymptomatic, with complete pulmonary re-expansion confirmed by CT imaging and chest radiograph (Figures 2A-2B).

## Discussion

*Salmonella *species are Gram-negative, facultative, anaerobic bacilli. Clinically, they are classified as typhoidal (*Salmonella t*yphi, *Salmonella *paratyphi) and non-typhoidal (*Salmonella* enteritidis, among others). Non-typhoidal *Salmonella *(NTS) infections commonly present as self-limited gastroenteritis; however, extraintestinal manifestations, including endovascular infections, osteomyelitis, and pleuropulmonary complications, occur in less than 1% of cases [2,3].

Our patient exhibited no gastrointestinal symptoms, and there was no known contact or familial history of recent enteric illness. Pleuropulmonary involvement in NTS infection is exceedingly rare, with approximately 40 cases described in the literature [4]. Carel et al. described the first case of NTS empyema in 1977 in a 60-year-old male patient, with a history of lung cancer treated with a long course of multiple antibiotics and intrapleural therapy with tetracycline without thoracotomy, contrary to the actual management of most empyemas requiring chest tube drainage and surgical management [5].

Most reported NTS empyema cases involve elderly or immunocompromised patients. Previous case reports have described multiple conditions that can influence the development of empyema, as reported in patients with HIV infection, malignancy, sickle cell disease, diabetes, chronic corticosteroid use, and advanced age [3-7].

Pleural involvement can result from hematogenous dissemination or direct spread from the spleen. Reticuloendothelial system (RES) colonization provides a reservoir for both typhoidal and non-typhoidal strains; reactivation of these dormant bacteria can facilitate the development of lung infection [4].

The absence of classic risk factors - such as HIV, malignancy, or diabetes - makes this case of *S. empyema *particularly unusual. Notably, the patient’s HbA1c and glucose levels were within normal limits. The presence of asthma, however, is a relevant factor; evidence suggests that it can increase the risk of empyema twofold. The pathophysiology likely involves a combination of impaired mucociliary function and increased bacterial susceptibility, particularly when the underlying respiratory disease is inadequately managed [8].

The progression of empyema thoracis is classified into three distinct stages based on the duration of infection and the inflammatory response. The exudative phase (Stage I) is marked by increased capillary permeability and the accumulation of sterile pleural fluid. This progresses to the fibrinopurulent phase (Stage II), defined by heavy fibrin deposition, bacterial invasion, and the formation of frank pus. The final organized phase (Stage III) involves fibroblast migration and the formation of a fibrous peel on the visceral pleura, which impairs lung re-expansion [9]. The clinical manifestations of NTS empyema in our patient are consistent with those described in the available literature for streptococcal empyema. Fever and general malaise predominated, frequently accompanied by respiratory symptoms, including cough and dyspnea [1,3-7].

Empyema treatment relies on prompt diagnosis, culture-guided antibiotic therapy, and effective drainage. While empirical therapy often involves ceftriaxone with metronidazole, our patient had previously received ceftriaxone and levofloxacin without significant improvement, warranting escalation. Surgical management via VATS or thoracotomy with pleurectomy is often necessary in complex or refractory cases [1]. The primary goals of surgical empyema management are adequate lung re-expansion and drainage of infected pleural fluid. VATS is often the preferred approach, given its documented benefits over thoracotomy, which include reduced blood loss, better pain management, shorter chest tube duration, and lower 30-day mortality. However, absolute contraindications to VATS, such as coagulopathy or the inability to tolerate single-lung ventilation (selective intubation), must be respected. In our patient, the presence of severe bronchospasm prohibited selective intubation, necessitating an immediate modification of the surgical approach to ensure patient safety [1,10,11].

Conversion to open thoracotomy is closely linked to the stage of empyema and the duration of symptoms, with conversion rates increasing significantly following a delay in surgical treatment exceeding 10 days from symptom onset. Other established risk factors include male gender, post-pneumonia etiology, and the isolation of Gram-negative bacteria. Based on the history gathered from the patient and his family, an estimated 12-day delay occurred between the onset of symptoms and the surgical intervention performed at our center, placing the patient at a high risk for conversion [12,13].

## Conclusions

Empyema caused by NTS is a rare clinical entity, particularly in patients without classical immunocompromising conditions. This case highlights that non-typhoidal *Salmonella *should be considered a potential causative pathogen in complicated pleural infections, even in the absence of gastrointestinal symptoms or recognized risk factors. Underlying respiratory diseases, such as asthma, may represent a contributory predisposition, although further research is warranted to better understand the association between asthma and increased susceptibility to invasive *Salmonella *infections, which will facilitate the development of adequate screening and prevention measures. Early recognition, prompt culture-directed antimicrobial therapy, and timely surgical intervention remain essential to achieve favorable outcomes. Additionally, this case underscores the importance of individualized surgical decision-making, as intraoperative patient factors may necessitate deviation from minimally invasive approaches to ensure safety and effective lung re-expansion.

## References

[1] Shen KR, Bribriesco A, Crabtree T (2017). The American Association for Thoracic Surgery consensus guidelines for the management of empyema. J Thorac Cardiovasc Surg.

[2] Harris JB, Ryan ET (2015). Enteric fever and other causes of fever and abdominal symptoms. Mandell, Douglas, and Bennett's Principles and Practice of Infectious Diseases, 8th Edition.

[3] Xaplanteri P, Assimakopoulos SF, Karachalios K (2016). Pleural empyema due to Salmonella enterica serovar Enteritidis in an immunocompetent elderly patient: a case report. JMM Case Rep.

[4] Crum NF (2005). Non-typhi Salmonella empyema: case report and review of the literature. Scand J Infect Dis.

[5] Carel RS, Schey G, Ma'ayan M, Bruderman I (1977). Salmonella empyema as a complication in malignant pleural effusion. Respiration.

[6] Nandan D, Jhavar L, Dewan V, Bhatt GC, Kaur N (2012). A case of empyema thoracic due to Salmonella typhi in 18-month-old child: an unusual cause. J Lab Physicians.

[7] Solanky D, Kwan B (2020). Nontyphoid Salmonella empyema in a patient with type 2 diabetes mellitus. J Glob Infect Dis.

[8] Liao WC, Lin CL, Shen TC, Tu CY, Hsia TC, Hsu WH (2022). Risk of pleural empyema in adult patients with asthma: a nationwide retrospective cohort study. Front Med (Lausanne).

[9] Higuchi M, Suzuki H (2020). Current status and prospect of medical and surgical management for thoracic empyema. Curr Chall Thorac Surg.

[10] Chung JH, Lee SH, Kim KT, Jung JS, Son HS, Sun K (2014). Optimal timing of thoracoscopic drainage and decortication for empyema. Ann Thorac Surg.

[11] Muhammad MI (2012). Management of complicated parapneumonic effusion and empyema using different treatment modalities. Asian Cardiovasc Thorac Ann.

[12] Lardinois D, Gock M, Pezzetta E, Buchli C, Rousson V, Furrer M, Ris HB (2005). Delayed referral and gram-negative organisms increase the conversion thoracotomy rate in patients undergoing video-assisted thoracoscopic surgery for empyema. Ann Thorac Sur.

[13] Hall RL, Partridge R, Venkatraman N, Wiselka M (2013). Invasive non-typhoidal Salmonella infection with multifocal seeding in an immunocompetent host: an emerging disease in the developed world. BMJ Case Rep.

